# Exploring households experiencing and at risk of homelessness: Linking homelessness case level data to Census 2021

**DOI:** 10.23889/ijpds.v8i2.2317

**Published:** 2023-09-14

**Authors:** Dilly Stephenson, Jen Hampton

**Affiliations:** 1 Office for National Statistics, Newport, United Kingdom

This paper describes linkage of Homelessness Case Level Collection (H-CLIC) data to Census 2021, allowing researchers to better understand households experiencing and at risk of homelessness. This fits within a wider portfolio of Better Outcomes Through Linked Data (BOLD) pilot projects, developing research possibilities and partnerships within the homelessness research sphere.

To mitigate impact from limited and lower quality variables, linkage was performed in two phases: individual-level linkage and associative matching. Individual-level linkage combined deterministic and probabilistic linkage methods, supported using Splink, with two stages of probabilistic linkage with differing extents of geographical blocking. Clerical-focused associative matching was employed following individual linkage, using UPRN and household make-up.

The project took a pipeline approach, with consideration to future-proofing the pipeline to facilitate annual updates to the H-CLIC spine. Also considered in detail are the quality implications of differing temporal data in linking dynamic H-CLIC data to static Census data.

The linkage resulted in construction of a research-ready dataset, which enables H-CLIC applicants to be joined to their Census records. To compensate for limited address information and support best use of processing resources, blocking was used to limit search spaces in the probabilistic linkage. Households with missing links between individual members were successfully identified and passed through associative matching methods. Though matching was successful, the remaining residuals were challenging to deal with.

Error introduced by the linkage methodology is presented via linkage quality metrics and bias analysis. Bias introduced by the linkage processing may be of particular concern, particularly considering coverage of these sample data. Within the fifty-three current Local Authorities included, a skew was observed toward smaller, southernly areas with lower homelessness rates.

The linked dataset enables research into households vulnerable to homelessness, their movements across England, and their exposure to repeated homelessness. The data facilitates research into this important topic, supporting evidence-based policymaking. Furthermore, such use is anticipated to encourage further local authorities to provide their data, broadening its reach and our understanding.

